# Methyl 4-[5-(4-fluoro­phen­yl)-4-(pyridin-4-yl)-1*H*-imidazol-2-ylsulfan­yl]butanoate

**DOI:** 10.1107/S1600536808016000

**Published:** 2008-06-07

**Authors:** Pierre Koch, Christiane Bäuerlein, Dieter Schollmeyer, Stefan Laufer

**Affiliations:** a Institute of Pharmacy, Department of Pharmaceutical and Medicinal Chemistry, Eberhard Karls University Tübingen, Auf der Morgenstelle 8, 72076 Tübingen, Germany; b Department of Organic Chemistry, Johannes Gutenberg University Mainz, Duesbergweg 10-14, 55099 Mainz, Germany

## Abstract

The title compound, C_19_H_18_FN_3_O_2_S, was synthesized in the course of studies on 2-alkyl­sufanylimidazoles as p38 mitogen-activated protein kinase inhibitors. The synthesis was achieved by nucleophilic substitution of 4-(4-fluoro­phen­yl)-5-(pyridin-4-yl)-1,3-dihydro­imidazole-2-thione with methyl 4-bromo­butanoate. The five-membered heterocycle makes dihedral angles of 32.4 (2) and 18.3 (2)° with the fluoro­phenyl and pyridinyl rings, respectively, indicating a low degree of conjugation between these rings. Intra­molecular C—H⋯N and inter­molecular N—H⋯N hydrogen bonds as well as C—H⋯π inter­actions seem to be effective in stabilization of the crystal structure.

## Related literature

Substituted imidazoles as small-mol­ecule inhibitors of p38 MAP kinase have been reviewed by Peifer *et al.* (2006 ▶) and Wagner & Laufer (2006 ▶). For the preparation of 4-(4-fluoro­phen­yl)-5-(pyridin-4-yl)-1,3-dihydro­imidazole-2-thione, see: Lantos *et al.* (1988 ▶). For related literature, see: Laufer, Striegel & Wagner (2002 ▶); Laufer, Wagner & Kotschenreuther (2002 ▶); Laufer & Koch (2008 ▶); Wang *et al.* (1998 ▶); Peifer *et al.* (2007 ▶).
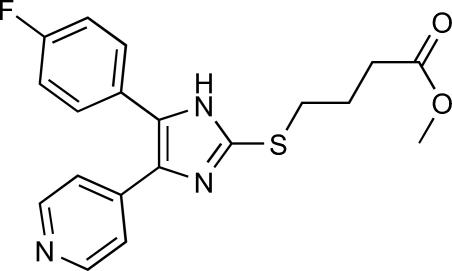


## Experimental

### 

#### Crystal data

C_19_H_18_FN_3_O_2_S
*M* *_r_* = 371.42
Orthorhombic, 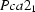

*a* = 18.494 (4) Å
*b* = 12.4367 (10) Å
*c* = 7.5255 (5) Å
*V* = 1730.9 (4) Å^3^
*Z* = 4
Cu *K*α radiation
μ = 1.92 mm^−1^
*T* = 193 (2) K
0.55 × 0.12 × 0.09 mm

#### Data collection

Enraf–Nonius CAD-4 diffractometer
Absorption correction: Gaussian (*PLATON*; Spek, 2003 ▶) *T*_min_ = 0.61, *T*_max_ = 0.85
3363 measured reflections
3086 independent reflections
2869 reflections with *I* > 2σ(*I*)
*R*_int_ = 0.051
3 standard reflections frequency: 60 min intensity decay: 5%

#### Refinement

*R*[*F* ^2^ > 2σ(*F* ^2^)] = 0.076
*wR*(*F* ^2^) = 0.188
*S* = 1.14
3086 reflections
236 parameters
1 restraint
H-atom parameters constrained
Δρ_max_ = 1.14 e Å^−3^
Δρ_min_ = −0.60 e Å^−3^
Absolute structure: Flack (1983 ▶), 1307 Friedel pairs
Flack parameter: −0.02 (3)

### 

Data collection: *CAD-4 Software* (Enraf–Nonius, 1989 ▶); cell refinement: *CAD-4 Software*; data reduction: *CORINC* (Dräger & Gattow, 1971 ▶); program(s) used to solve structure: *SIR97* (Altomare *et al.*, 1999 ▶); program(s) used to refine structure: *SHELXL97* (Sheldrick, 2008 ▶); molecular graphics: *PLATON* (Spek, 2003 ▶); software used to prepare material for publication: *PLATON*.

## Supplementary Material

Crystal structure: contains datablocks I, global. DOI: 10.1107/S1600536808016000/bx2140sup1.cif

Structure factors: contains datablocks I. DOI: 10.1107/S1600536808016000/bx2140Isup2.hkl

Additional supplementary materials:  crystallographic information; 3D view; checkCIF report

## Figures and Tables

**Table 1:** Hydrogen-bond geometry (Å, °)

*D*—H⋯*A*	*D*—H	H⋯*A*	*D*⋯*A*	*D*—H⋯*A*
N5—H5⋯N17^i^	0.90	1.95	2.849 (4)	174

Symmetry code: (i) 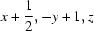.

**Table 2:** Nonconventional C—H⋯*X* contacts (Å, °)

C–H⋯A	C–H	H⋯A	C–H⋯A	C⋯A
C13–H13*B*⋯*Cg*1^ii^	0.98	2.65	156	3.566 (6)
C7–H7*A*⋯N2	0.99	2.57	100	2.910 (5)

Symmetry code: (ii) 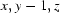. *Cg*1 is the centroid of the C20–C25 ring.

**Fig. 1.**
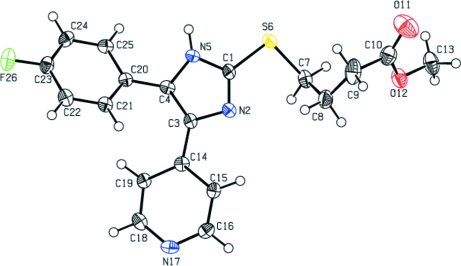
View of compound I. Displacement ellipsoids are drawn at the 50% probability level. H atoms are depicted as circles of arbitrary size.

**Fig. 2.**
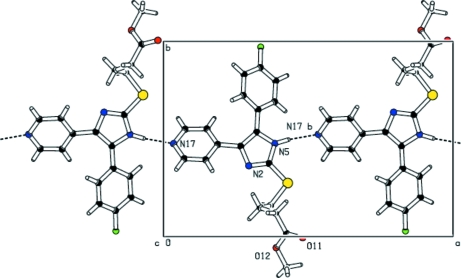
Part of the crystal packing of compound I. The hydrogen bond is shown with dashed lines. View along c axis. N17_b:x + 1/2, 1 - y, z

## supplementary crystallographic information

### Comment

The title compound was prepared in the course of our studies on 2-alkylsulfanyl-4-(4-fluorophenyl)-5-pyridinyl imidazoles as p38 mitogen-activated protein (MAP) kinase inhibitors. The p38 MAP kinase plays a central role for the biosynthesis and release of pro-inflammatory cytokines like TNF-α and IL-1β. Inhibition of p38 MAP kinase is therefore a promising therapeutic strategy for the treatment of inflammatory disorders like psoriasis, inflammatory bowel disease and rheumatoid arthritis. The fundamental SAR for the class of pyridinyl imidazole derivative as p38 MAP kinase inhibitors can be exemplified by the way SB203580 binds to the protein (Wang *et al.*, 1998). There is a crucial hydrogen bond between the pyridin-4-yl moiety and Met109 of the enzyme. The 4-fluorophenyl ring binds to the hydrophobic region I, mainly gaining selectivity. Another possible ligand-protein interaction is a hydrogen bond between Lys53 and N3 of the imidazole core (Peifer *et al., *2007).

The analysis of the crystal structure of methyl 4-(5-(4-fluorophenyl)-4-(pyridin-4-yl)-1*H*-imidazol-2-ylthio)butanoate (I) is shown in Figure 1. The crystal packing (Figure 2) shows that N5—H5 of the imidazole ring forms an intermolecular N–H···N hydrogen bond to pyridine (N17). The length of the hydrogen bond is 1.95Å (Table 1). Non-conventional C—H···*X* H-bonds are also present in addition to intermolecular N—H···N hydrogen interactions (Table 2).

### Experimental

To a stirred solution of 4-(4-fluorophenyl)-5-(pyridin-4-yl)-1,3-dihydroimidazole-2-thione (0.74 mmol) and potassium *tert*-butoxide (0.77 mmol) in dry methanol (15 ml) was added under argon atmosphere after 15 min metyl 4-bromobutanoate (0.77 mmol). The solution was heated for 1 h to reflux temperature. After extraction with water and ethyl acetate the organic layer was washed twice with water, dried over sodium sulfate and evaporated under reduced pressure. The crude product was purified by flash chromatography (silica gel, dichloromethane - ethyl acetate 1:1 to 2:3) to yield methyl 4-(5-(4-fluorophenyl)-4-(pyridin-4-yl)-1*H*-imidazol-2-ylthio)butanoate (I) (49%) as a colorless solid. Compound I was crystallized from methanol.

### Refinement

Hydrogen atoms attached to carbons were placed at calculated positions with C—H = 0.95Å (aromatic) or 0.98–0.99 Å (*sp*^3^ C-atom). H-atom bonded to N5 was located from a difference Fourier map (N—H = 0.9 Å). All H atoms were refined in the riding-model approximation with isotropic displacement parameters (set at 1.2–1.5 times of the *U*_eq_ of the parent atom).

### Crystal data

**Table:**

C_19_H_18_FN_3_O_2_S	*F*_000_ = 776
*M**_r_* = 371.42	*D*_x_ = 1.425 Mg m^−^^3^
Orthorhombic, *P**c**a*2_1_	Cu *K*α radiation λ = 1.54178 Å
Hall symbol: P 2c -2ac	Cell parameters from 25 reflections
*a* = 18.494 (4) Å	θ = 31–44º
*b* = 12.4367 (10) Å	µ = 1.92 mm^−^^1^
*c* = 7.5255 (5) Å	*T* = 193 (2) K
*V* = 1730.9 (4) Å^3^	Needle, colourless
*Z* = 4	0.55 × 0.12 × 0.09 mm

### Data collection

**Table:**

Enraf–Nonius CAD-4 diffractometer	*R*_int_ = 0.051
Monochromator: graphite	θ_max_ = 70.0º
*T* = 193(2) K	θ_min_ = 3.6º
ω/2θ scans	*h* = −22→22
Absorption correction: gaussian(PLATON; Spek, 2003)	*k* = −15→15
*T*_min_ = 0.61, *T*_max_ = 0.85	*l* = −7→9
3363 measured reflections	3 standard reflections
3086 independent reflections	every 60 min
2869 reflections with *I* > 2σ(*I*)	intensity decay: 5%

### Refinement

**Table:**

Refinement on *F*^2^	Hydrogen site location: inferred from neighbouring sites
Least-squares matrix: full	H-atom parameters constrained
*R*[*F*^2^ > 2σ(*F*^2^)] = 0.076	*w* = 1/[σ^2^(*F*_o_^2^) + (0.1374*P*)^2^] where *P* = (*F*_o_^2^ + 2*F*_c_^2^)/3
*wR*(*F*^2^) = 0.188	(Δ/σ)_max_ < 0.001
*S* = 1.14	Δρ_max_ = 1.14 e Å^−^^3^
3086 reflections	Δρ_min_ = −0.60 e Å^−^^3^
236 parameters	Extinction correction: none
1 restraint	Absolute structure: Flack (1983), 1307 Friedel pairs
Primary atom site location: structure-invariant direct methods	Flack parameter: −0.02 (3)
Secondary atom site location: difference Fourier map	

### Special details

**Table:**

Geometry. All e.s.d.'s (except the e.s.d. in the dihedral angle between two l.s. planes) are estimated using the full covariance matrix. The cell e.s.d.'s are taken into account individually in the estimation of e.s.d.'s in distances, angles and torsion angles; correlations between e.s.d.'s in cell parameters are only used when they are defined by crystal symmetry. An approximate (isotropic) treatment of cell e.s.d.'s is used for estimating e.s.d.'s involving l.s. planes.
Refinement. Refinement of *F*^2^ against ALL reflections. The weighted *R*-factor *wR* and goodness of fit *S* are based on *F*^2^, conventional *R*-factors *R* are based on *F*, with *F* set to zero for negative *F*^2^. The threshold expression of *F*^2^ > σ(*F*^2^) is used only for calculating *R*-factors(gt) *etc*. and is not relevant to the choice of reflections for refinement. *R*-factors based on *F*^2^ are statistically about twice as large as those based on *F*, and *R*- factors based on ALL data will be even larger.

### Fractional atomic coordinates and isotropic or equivalent isotropic displacement parameters (Å^2^)

**Table:**

	*x*	*y*	*z*	*U*_iso_*/*U*_eq_	
C1	0.3643 (2)	0.3750 (3)	0.3172 (5)	0.0204 (8)	
N2	0.29502 (16)	0.3630 (3)	0.3227 (4)	0.0203 (7)	
C3	0.26668 (19)	0.4629 (3)	0.2819 (5)	0.0179 (7)	
C4	0.32215 (19)	0.5351 (3)	0.2524 (5)	0.0183 (8)	
N5	0.38455 (15)	0.4764 (2)	0.2738 (4)	0.0173 (6)	
H5	0.4314	0.4967	0.2711	0.021*	
S6	0.42920 (5)	0.27420 (8)	0.35250 (18)	0.0298 (3)	
C7	0.3694 (2)	0.1589 (3)	0.3710 (7)	0.0300 (9)	
H7A	0.3312	0.1650	0.2796	0.036*	
H7B	0.3975	0.0929	0.3456	0.036*	
C8	0.3338 (2)	0.1474 (4)	0.5527 (8)	0.0371 (12)	
H8A	0.2967	0.0901	0.5463	0.044*	
H8B	0.3089	0.2155	0.5825	0.044*	
C9	0.3870 (3)	0.1202 (4)	0.7011 (8)	0.0451 (13)	
H9A	0.4239	0.1778	0.7061	0.054*	
H9B	0.3604	0.1214	0.8153	0.054*	
C10	0.4252 (2)	0.0150 (4)	0.6867 (7)	0.0360 (11)	
O11	0.4808 (2)	−0.0057 (4)	0.7602 (7)	0.0621 (12)	
O12	0.38975 (17)	−0.0571 (2)	0.5875 (5)	0.0368 (8)	
C13	0.4252 (3)	−0.1576 (4)	0.5589 (7)	0.0413 (12)	
H13A	0.4738	−0.1447	0.5115	0.062*	
H13B	0.3973	−0.2004	0.4737	0.062*	
H13C	0.4288	−0.1965	0.6717	0.062*	
C14	0.1875 (2)	0.4721 (3)	0.2746 (5)	0.0189 (7)	
C15	0.14573 (19)	0.3934 (3)	0.3609 (6)	0.0225 (7)	
H15	0.1684	0.3374	0.4260	0.027*	
C16	0.07186 (19)	0.3987 (3)	0.3501 (7)	0.0267 (8)	
H16	0.0447	0.3439	0.4076	0.032*	
N17	0.03515 (16)	0.4759 (3)	0.2637 (6)	0.0270 (8)	
C18	0.0753 (2)	0.5502 (3)	0.1816 (6)	0.0244 (9)	
H18	0.0509	0.6056	0.1186	0.029*	
C19	0.14979 (19)	0.5514 (3)	0.1825 (5)	0.0201 (7)	
H19	0.1753	0.6061	0.1207	0.024*	
C20	0.32620 (18)	0.6506 (3)	0.2109 (5)	0.0185 (8)	
C21	0.2750 (2)	0.7236 (3)	0.2741 (6)	0.0217 (8)	
H21	0.2373	0.6988	0.3493	0.026*	
C22	0.2782 (2)	0.8313 (3)	0.2294 (6)	0.0261 (9)	
H22	0.2423	0.8801	0.2704	0.031*	
C23	0.3342 (2)	0.8664 (3)	0.1244 (6)	0.0274 (9)	
C24	0.3875 (2)	0.7984 (4)	0.0657 (6)	0.0267 (9)	
H24	0.4265	0.8252	−0.0032	0.032*	
C25	0.38349 (19)	0.6910 (3)	0.1081 (6)	0.0220 (8)	
H25	0.4201	0.6434	0.0672	0.026*	
F26	0.33578 (16)	0.9714 (2)	0.0773 (4)	0.0405 (7)	

### Atomic displacement parameters (Å^2^)

**Table:**

	*U*^11^	*U*^22^	*U*^33^	*U*^12^	*U*^13^	*U*^23^
C1	0.0269 (17)	0.0264 (18)	0.008 (2)	0.0036 (14)	−0.0006 (13)	0.0032 (14)
N2	0.0239 (14)	0.0260 (15)	0.0110 (19)	0.0029 (11)	0.0001 (12)	−0.0007 (12)
C3	0.0263 (18)	0.0242 (17)	0.0031 (17)	0.0023 (13)	0.0009 (14)	−0.0015 (14)
C4	0.0211 (16)	0.032 (2)	0.0019 (18)	0.0020 (14)	0.0004 (13)	−0.0021 (14)
N5	0.0177 (13)	0.0271 (16)	0.0072 (15)	0.0012 (11)	−0.0002 (12)	−0.0022 (12)
S6	0.0229 (5)	0.0303 (5)	0.0362 (7)	0.0068 (3)	0.0024 (4)	0.0086 (4)
C7	0.037 (2)	0.0238 (18)	0.029 (3)	0.0044 (15)	−0.005 (2)	0.0005 (17)
C8	0.039 (2)	0.033 (2)	0.039 (3)	0.0064 (18)	0.011 (2)	0.013 (2)
C9	0.078 (4)	0.033 (2)	0.024 (3)	0.004 (2)	0.005 (3)	0.002 (2)
C10	0.045 (3)	0.037 (2)	0.026 (3)	−0.0026 (18)	0.002 (2)	0.004 (2)
O11	0.059 (2)	0.061 (2)	0.067 (3)	0.009 (2)	−0.027 (2)	−0.019 (2)
O12	0.0440 (17)	0.0313 (16)	0.035 (2)	0.0030 (12)	−0.0122 (15)	0.0029 (15)
C13	0.060 (3)	0.035 (2)	0.029 (3)	0.008 (2)	−0.014 (2)	0.002 (2)
C14	0.0255 (17)	0.0277 (17)	0.0036 (17)	−0.0002 (14)	0.0021 (14)	−0.0047 (14)
C15	0.0294 (17)	0.0239 (16)	0.014 (2)	0.0004 (14)	0.0036 (18)	−0.0007 (15)
C16	0.0281 (18)	0.0265 (18)	0.025 (2)	−0.0021 (14)	0.0060 (18)	−0.0044 (19)
N17	0.0193 (14)	0.0349 (18)	0.027 (2)	−0.0003 (13)	−0.0008 (13)	−0.0080 (15)
C18	0.033 (2)	0.031 (2)	0.010 (2)	0.0058 (15)	−0.0052 (16)	−0.0029 (17)
C19	0.0266 (18)	0.0275 (17)	0.006 (2)	−0.0008 (14)	0.0003 (14)	−0.0004 (15)
C20	0.0224 (17)	0.0276 (18)	0.0055 (18)	−0.0017 (13)	−0.0034 (13)	−0.0007 (14)
C21	0.0266 (18)	0.031 (2)	0.0076 (18)	0.0008 (14)	−0.0018 (15)	0.0005 (15)
C22	0.034 (2)	0.0285 (19)	0.016 (2)	0.0043 (16)	−0.0011 (16)	−0.0031 (16)
C23	0.037 (2)	0.0259 (19)	0.020 (2)	−0.0043 (15)	−0.0059 (17)	0.0033 (16)
C24	0.031 (2)	0.036 (2)	0.013 (2)	−0.0078 (16)	−0.0001 (15)	0.0056 (17)
C25	0.0221 (17)	0.0320 (19)	0.012 (2)	−0.0004 (14)	0.0006 (15)	−0.0026 (16)
F26	0.0596 (17)	0.0272 (12)	0.0346 (18)	−0.0027 (11)	0.0013 (13)	0.0072 (11)

### Geometric parameters (Å, °)

**Table:**

C1—N2	1.290 (5)	C13—H13B	0.9800
C1—N5	1.356 (5)	C13—H13C	0.9800
C1—S6	1.756 (4)	C14—C19	1.392 (5)
N2—C3	1.384 (5)	C14—C15	1.405 (5)
C3—C4	1.381 (5)	C15—C16	1.370 (5)
C3—C14	1.470 (5)	C15—H15	0.9500
C4—N5	1.375 (5)	C16—N17	1.344 (6)
C4—C20	1.471 (5)	C16—H16	0.9500
N5—H5	0.9032	N17—C18	1.336 (6)
S6—C7	1.816 (4)	C18—C19	1.378 (5)
C7—C8	1.525 (7)	C18—H18	0.9500
C7—H7A	0.9900	C19—H19	0.9500
C7—H7B	0.9900	C20—C21	1.395 (5)
C8—C9	1.527 (8)	C20—C25	1.405 (5)
C8—H8A	0.9900	C21—C22	1.383 (5)
C8—H8B	0.9900	C21—H21	0.9500
C9—C10	1.491 (6)	C22—C23	1.374 (6)
C9—H9A	0.9900	C22—H22	0.9500
C9—H9B	0.9900	C23—F26	1.354 (5)
C10—O11	1.196 (6)	C23—C24	1.371 (6)
C10—O12	1.339 (6)	C24—C25	1.375 (6)
O12—C13	1.427 (6)	C24—H24	0.9500
C13—H13A	0.9800	C25—H25	0.9500
			
N2—C1—N5	113.0 (3)	H13A—C13—H13B	109.5
N2—C1—S6	126.2 (3)	O12—C13—H13C	109.5
N5—C1—S6	120.7 (3)	H13A—C13—H13C	109.5
C1—N2—C3	105.3 (3)	H13B—C13—H13C	109.5
C4—C3—N2	109.8 (3)	C19—C14—C15	116.6 (3)
C4—C3—C14	133.1 (3)	C19—C14—C3	124.9 (3)
N2—C3—C14	117.1 (3)	C15—C14—C3	118.4 (3)
N5—C4—C3	105.0 (3)	C16—C15—C14	119.2 (4)
N5—C4—C20	120.0 (3)	C16—C15—H15	120.4
C3—C4—C20	134.9 (3)	C14—C15—H15	120.4
C1—N5—C4	106.9 (3)	N17—C16—C15	124.5 (4)
C1—N5—H5	122.1	N17—C16—H16	117.8
C4—N5—H5	130.9	C15—C16—H16	117.8
C1—S6—C7	99.14 (18)	C18—N17—C16	115.9 (3)
C8—C7—S6	114.0 (3)	N17—C18—C19	124.1 (4)
C8—C7—H7A	108.8	N17—C18—H18	117.9
S6—C7—H7A	108.8	C19—C18—H18	117.9
C8—C7—H7B	108.8	C18—C19—C14	119.7 (4)
S6—C7—H7B	108.8	C18—C19—H19	120.2
H7A—C7—H7B	107.7	C14—C19—H19	120.2
C7—C8—C9	113.4 (4)	C21—C20—C25	117.8 (4)
C7—C8—H8A	108.9	C21—C20—C4	121.9 (3)
C9—C8—H8A	108.9	C25—C20—C4	120.3 (3)
C7—C8—H8B	108.9	C22—C21—C20	121.3 (4)
C9—C8—H8B	108.9	C22—C21—H21	119.4
H8A—C8—H8B	107.7	C20—C21—H21	119.4
C10—C9—C8	116.6 (4)	C23—C22—C21	118.7 (4)
C10—C9—H9A	108.2	C23—C22—H22	120.7
C8—C9—H9A	108.2	C21—C22—H22	120.7
C10—C9—H9B	108.2	F26—C23—C24	119.7 (4)
C8—C9—H9B	108.2	F26—C23—C22	118.3 (4)
H9A—C9—H9B	107.3	C24—C23—C22	122.1 (4)
O11—C10—O12	122.4 (5)	C23—C24—C25	119.1 (4)
O11—C10—C9	124.3 (5)	C23—C24—H24	120.5
O12—C10—C9	113.3 (4)	C25—C24—H24	120.5
C10—O12—C13	116.5 (4)	C24—C25—C20	121.0 (4)
O12—C13—H13A	109.5	C24—C25—H25	119.5
O12—C13—H13B	109.5	C20—C25—H25	119.5
			
N5—C1—N2—C3	−0.5 (4)	N2—C3—C14—C15	19.8 (5)
S6—C1—N2—C3	−178.3 (3)	C19—C14—C15—C16	0.1 (6)
C1—N2—C3—C4	−0.2 (4)	C3—C14—C15—C16	−177.7 (4)
C1—N2—C3—C14	178.7 (3)	C14—C15—C16—N17	−1.2 (7)
N2—C3—C4—N5	0.8 (4)	C15—C16—N17—C18	1.3 (7)
C14—C3—C4—N5	−177.8 (4)	C16—N17—C18—C19	−0.4 (6)
N2—C3—C4—C20	−177.7 (4)	N17—C18—C19—C14	−0.5 (6)
C14—C3—C4—C20	3.6 (7)	C15—C14—C19—C18	0.7 (6)
N2—C1—N5—C4	1.0 (4)	C3—C14—C19—C18	178.3 (3)
S6—C1—N5—C4	179.0 (3)	N5—C4—C20—C21	−146.2 (4)
C3—C4—N5—C1	−1.1 (4)	C3—C4—C20—C21	32.2 (7)
C20—C4—N5—C1	177.7 (4)	N5—C4—C20—C25	32.5 (5)
N2—C1—S6—C7	5.7 (4)	C3—C4—C20—C25	−149.2 (4)
N5—C1—S6—C7	−172.0 (3)	C25—C20—C21—C22	3.4 (6)
C1—S6—C7—C8	−80.0 (3)	C4—C20—C21—C22	−177.9 (4)
S6—C7—C8—C9	−67.3 (4)	C20—C21—C22—C23	−1.7 (6)
C7—C8—C9—C10	−63.6 (6)	C21—C22—C23—F26	178.2 (4)
C8—C9—C10—O11	158.8 (6)	C21—C22—C23—C24	−1.2 (7)
C8—C9—C10—O12	−23.6 (6)	F26—C23—C24—C25	−177.2 (4)
O11—C10—O12—C13	−6.0 (8)	C22—C23—C24—C25	2.2 (7)
C9—C10—O12—C13	176.3 (4)	C23—C24—C25—C20	−0.4 (6)
C4—C3—C14—C19	20.7 (7)	C21—C20—C25—C24	−2.4 (6)
N2—C3—C14—C19	−157.8 (4)	C4—C20—C25—C24	178.9 (4)
C4—C3—C14—C15	−161.6 (4)		

### Hydrogen-bond geometry (Å, °)

**Table:**

*D*—H···*A*	*D*—H	H···*A*	*D*···*A*	*D*—H···*A*
N5—H5···N17^i^	0.90	1.95	2.849 (4)	174

Symmetry codes: (i) *x*+1/2, −*y*+1, *z*.

### Table 2 Nonconventional C–H···X contacts (Å, °).

**Table:**

C–H···A	C–H	H···A	C–H···A	C···A
C13–H13B···Cg1^ii^	0.98	2.65	156	3.566 (6)
C7–H7A···N2	0.99	2.57	100	2.910 (5)

Symmetry code: (ii) x, y-1, z. Cg1 is the centroid of the C20–C25 ring.
